# Protective Role of Quercetin in Carbon Tetrachloride Induced Toxicity in Rat Brain: Biochemical, Spectrophotometric Assays and Computational Approach

**DOI:** 10.3390/molecules26247526

**Published:** 2021-12-12

**Authors:** Seema Zargar, Tanveer A. Wani

**Affiliations:** 1 Department of Biochemistry, College of Science, King Saud University, Riyadh 11495, Saudi Arabia; 2 Department of Pharmaceutical Chemistry, College of Pharmacy, King Saud University, Riyadh 11451, Saudi Arabia; twani@ksu.edu.sa

**Keywords:** quercetin, CCL4, neurotoxicity, VirtualToxLab, oxidative stress markers

## Abstract

Carbon tetrachloride (CCL4) induces oxidative stress by free radical toxicities, inflammation, and neurotoxicity. Quercetin (Q), on the other hand, has a role as anti-inflammatory, antioxidant, antibacterial, and free radical-scavenging. This study explored protection given by quercetin against CCL4 induced neurotoxicity in rats at given concentrations. Male Wistar rats were divided into four groups Group C: control group; Group CCL4: given a single oral dose of 1 mL/kg bw CCL4; Group Q: given a single i.p injection of 100 mg/kg bw quercetin; and Group Q + CCL4: given a single i.p injection of 100 mg/kg bw quercetin before two hours of a single oral dose of 1 mL/kg bw CCL4. The results from brain-to-body weight ratio, morphology, lipid peroxidation, brain urea, ascorbic acid, reduced glutathione, sodium, and enzyme alterations (aspartate aminotransferase (AST), alanine aminotransferase (ALT), catalase, and superoxide dismutase) suggested alterations by CCL4 and a significant reversal of these parameters by quercetin. In silico analysis of quercetin with various proteins was conducted to understand the molecular mechanism of its protection. The results identified by BzScore4 D showed moderate binding between quercetin and the following receptors: glucocorticoids, estrogen beta, and androgens and weak binding between quercetin and the following proteins: estrogen alpha, Peroxisome proliferator-activated receptors (PPARγ), Herg k+ channel, Liver x, mineralocorticoid, progesterone, Thyroid α, and Thyroid β. Three-dimensional/four-dimensional visualization of binding modes of quercetin with glucocorticoids, estrogen beta, and androgen receptors was performed. Based on the results, a possible mechanism is hypothesized for quercetin protection against CCL4 toxicity in the rat brain.

## 1. Introduction

Exposure to toxic chemicals, environmental changes, and drugs can cause harmful effects and injuries through the metabolic production of reactive oxygen species (ROS). The description of oxidative stress is an imbalance between ROS production and antioxidant defense, which causes cell damage at high levels. The ROS and their pathophysiological effects depend on the concentration, type, and specific production site. When ROS are at a high level, they react with DNA, proteins and cell membrane, and other molecules, causing cellular damage and producing other more reactive radicals [1]. In addition, the formation of ROS leads to DNA strand breaks and oxidative DNA damage that induce changes in mRNA expression of DNA damage responsive genes [2].

Carbon tetrachloride (CCL4), a colorless, transparent, heavy, and non-flammable industrial liquid, is widely used to induce free radical toxicity in various experimental animal tissues such as kidneys, heart, liver, lung, testis, brain, and blood [3]. Exposure to CCL4 initiates a complex process for resistance to toxicity and production of free radicals to metabolize CCL4, leading to further oxidative stress, which participates in the initiation and progression of brain injury [4]. The resulting oxidative stress leads to DNA fragmentation and produces a significant interconnected change of cellular metabolism and destruction of the cells by lipid peroxidation [4]. Acute and harmful tissue injuries are induced by CCL4 metabolites, reactive metabolic trichloromethyl radicals (CCl3), and peroxy trichloromethyl radicals (OOCCl3). The single hepatotoxic dose produces more intense free-radical stress in the brain than in the liver [5]. These free radicals can covalently bind to macromolecules such as lipids, proteins, and nucleic acids present in the brain [6]. Previous studies showed that CCL4-induction caused an observed reduction in p53, a tumor suppressor gene expression [7]. The antioxidant mechanism prevents cells in the G phase of the cell cycle and gives additional time for DNA repair, while severe DNA damage triggers apoptosis, a life-threatening condition [7].

Flavonoids are polyphenolic compounds that play an essential role in free radical detoxification. These polyphenolic compounds are found in vegetables, fruits, and medicinal plants. Quercetin in plants exists as either a free (aglycone) or conjugated with carbohydrates (quercetin glycosides) and alcohols (quercetin methyl ethers). It was reported that quercetin is an anti-inflammatory, antioxidant, antibacterial, radical-scavenging, antiviral, gastroprotective, and immune-modulator, and is used to treat cardiovascular diseases and obesity [8,9]. Quercetin is abundantly present in apples, berries, onions, capers, broccoli, tea, and red wine. Quercetin is reported to protect against CCL4 induced hepatotoxicity by inhibiting Toll-like receptor 2 (TLR2), TLR4 activation, and mitogen-activated protein kinase (MAP Kinase) phosphorylation. These, in turn, inactivate nuclear factor kappa B (NF-ĸB) and the inflammatory cytokines in livers of the CCL4-treated animals. Quercetin is reported to protect against brain injury in mice through TLR2/4 and MAPK/NF-ĸB pathway [10]. A high concentration of quercetin metabolites is present in the brain after several hours of quercetin administration [11].

An intraperitoneal dose of quercetin 10 mg/kg body weight in rats before two hours of acrylamide assault resulted in diminutive acrylamide mediated neurotoxicity. Quercetin treatment leads to decreased dopamine, interferon-γ, and 8-hydroxyguanosine levels and the restoration of serotonin levels [12]. In addition, quercetin can increase the body′s antioxidant activity by regulating glutathione (GSH) levels. The GSH is a central component of a natural defense mechanism of the body against oxidative stress. The superoxide dismutase (SOD) quickly captures O^2−^ and transforms it into hydrogen peroxide H_2_O_2_. This enzyme further catalyzes the decomposition of H_2_O_2_ to the non-toxic H_2_O. This reaction requires GSH as a hydrogen donor. Quercetin in several studies was found to induce reduced glutathione (GSH) synthesis. One study reported that the p53 penetrates in the mechanism of cell response to quercetin through modulation of glutathione-related enzyme expression [13,14]. ROS and reactive nitrogen species (RNS) are produced continuously in the body by oxidative metabolism, mitochondrial bioenergetics, and immune function that can cause potential biological damage [15]. As a flawed anti-oxidative system favors the accumulation of free radicals due to a decrease in the activity of antioxidant enzymes, quercetin may find application in the prevention of neurological disorders due to its neuroprotective effects. The present study evaluated the toxicity of CCL4 in rat brains and investigated whether quercetin can protect against the damage caused by it.

The VirtualToxLab can predict the toxic potential of the tested compound, e.g., endocrine and metabolic disruption, some aspects of carcinogenicity, and cardiotoxicity by simulation and quantification of their interactions towards a series of proteins suspected to trigger mentioned adverse effects [16]. This tool follows an automated protocol and calculates the binding affinity of the investigated compound to selected proteins. It is beneficial to understand that interaction mechanism at the molecular level and estimate the toxic potential of the studied drugs. The VirtualToxLab™ was used to study the interaction between quercetin and various proteins to understand the molecular basis of the protective potential of quercetin against carbon tetrachloride toxicity on rat brains.

## 2. Materials and Methods

### 2.1. Chemicals

All the reagents and chemicals, including quercetin and CCL4, were obtained from Sigma Chemical C., St Louis, MO, USA. All the kits for enzymatic analysis were purchased from United Diagnostics Industry (Dammam, Saudi Arabia).

### 2.2. Animals

Male Wistar rats 8–12 weeks of age, weighing 80–90 g (*n* = 24), were obtained from the Animal House Facility of King Saud University, Riyadh, Saudi Arabia. The animal ethics committee approved the study of King Saud University (approval no. KSU-SE-21-05). Rats were housed at 23–25 °C and 55–60% ambient humidity on a 12:12 h light/dark cycle and were fed a regular diet with fresh drinking water daily.

### 2.3. Experimental Design

Group C: control group; Group CCL4: exposure to CCL4; Group Q: exposure to quercetin only; Group Q + CCL4: treated with both quercetin and CCL4. Each group consisted of six animals. Sunflower oil (vehicle) was used to dissolve CCL4 and then administered to specific groups of animals. To the control group, only the sunflower oil was administered. Due to CCL4’s non-polar nature and high volatility, it was necessary to dissolve it in sunflower oil (3:1) to maintain a consistent, effective dose. Neurotoxicity in mice occurs with a single dose of CCL4 (1 mL/kg bw) [5]. To the CCL4 Group, the CCL4 dose was administered by oral gavage. To the quercetin-only group (Group Q), quercetin was given as a single dose of i.p injection of quercetin (100 mg/kg bw) [17,18,19]. In the combination group, quercetin (100 mg/kg bw) was administered 2 h before the assault by CCL4 (1 mL/kg bw). The Cmax of quercetin is 2–3 h; therefore, quercetin was administered to obtain protective concentration levels of quercetin in systemic circulation before the CCL4 assault [10]. The animals were sacrificed by carbon dioxide asphyxiation after 24 h of their respective treatments bw) [17,18,19,20]. The brains of the studied groups were removed and processed immediately for biochemical assays. The harvested brains were weighed immediately for determining the organ/body weight ratio. The harvested brains were homogenized in 1X PBS, and slices from the cortex were cut and stored in formalin for histopathology.

The samples were processed as follows: fixing the specimens in a 10% neutral buffered formalin solution (Loba Chemie, Colaba, India) block preparation in paraffin (Leica Biosystems, Wetzlar, Germany) cutting sections of 5–6 μm thick, and the sections stained with hematoxylin-eosin stain (Leica Biosystems, Wetzlar, Germany). The sections were photographed and analyzed using an electron microscope (Leica Biosystems, Wetzlar, Germany) by an expert pathologist who was not informed about the sample assigned to the experimental groups.

### 2.4. Brain Enzymes

Alanine aminotransferase (ALT) and aspartate aminotransferase (AST) were measured with UV-kinetic diagnostic kits according to the kit protocol of the manufacturer (United Diagnostics Industry (UDI, Dammam, Saudi Arabia). The method was based on the oxidation of NADH, and the rate of decrease in absorbance at 340 nm is proportional to the ALT activity of the sample. ALT and AST reagents were reconstituted with 3 mL of distilled water, then 1000 µLof reconstituted reagent were pre-warmed at 37 °C for 2 min followed by mixing with 100 µLof the sample. The mixture was allowed to stand for 60 s for temperature equilibrium. ALT absorbance was measured every 60 s within 3 min at 340 nm against distilled water, and AST absorbance was measured every 60 s within a 2-min interval at 340 nm against distilled water. Eventually, ΔA/min was determined. Each unit of AST and ALT enzyme activity was defined as micromoles of NADH decomposed per minute using a molar absorbance of 6.22 × 10^3^ × M^−1^ Cm^−1^.

Superoxide dismutase was determined by the method of Nishikimi et al. [21]. Briefly, the reaction mixture contained 0.1 mL of sodium pyrophosphate (0.1 mM), 0.1 mL of NBT (0.3 mM), 0.1 mL of NADH (0.47 mM), 0.05 mL of PMS (0.93 µM), and 0.1 mL of enzyme (homogenized brain tissue) in a total volume of 1 mL. The rate of change of absorbance was measured at 560 nm. Values were expressed as units of enzyme min^−1^ mg protein. Catalase activity was estimated in the whole homogenized brain tissue by the method of Aebie, 1984 [22]. The reaction mixture in a total volume of 3 mL contained 0.4 M sodium phosphate buffer pH 7.2, 1.2 mL of H_2_O_2_, and a suitably diluted enzyme. The reaction was started by adding H_2_O_2_ and reading the change in absorbance at 240 nm for two minutes. One unit of CAT activity was defined as micromoles of H_2_O_2_ decomposed per min using molar absorbance of H_2_O_2_ (43.6 M^−1^ Cm^−1^).

### 2.5. Measurement of Brain Sodium

Measurement of brain sodium was done with a sodium estimation kit procured from United Diagnostics Industry (UDI, Dammam, KSA), as per the kit protocol procedures. Na⁺/K⁺-ATPase is an enzyme found in the cell′s membrane. It performs several functions in cell physiology. The assay activates the B-galactosidase enzyme by the sodium present in the sample and the consequent enzymatic transformation of o-nitrophenyl-β, D-glactopyranoside (o-NPG) into o-nitrophenol, and galactose. The o-nitrophenol formed was kinetically measured at 405 nm against distilled water every 20 s for two minutes as per the kit protocol. One unit of sodium level was defined as micromoles of o-NPG (o-nitrophenyl-β, D-glactopyranoside) decomposed per minute using molar absorbance of 18.75 × 10^3^ M^−1^ Cm^−1^.

### 2.6. Measurement of Brain Urea

Brain Urea was calculated using GLDH—UV-kinetic diagnostic kit with the SEMI MICRO METHOD from United Diagnostics Industry (UDI, Dammam, Saudi Arabia). Urea is a primary end product of protein nitrogen metabolism. The urea reagent was reconstituted with 3 mL of distilled water, then 1000 µL of reconstituted reagent was pre-warmed at 37 °C for 2 min followed by mixing with 10 µL of the sample. Absorbance was measured every 30 s for 90 s at 340 nm against distilled water, and eventually, ΔA/min was determined. One urea unit was defined as micromoles of NADH decomposed per minute using molar absorbance of 6.22 × 10^3^ × M^−1^ Cm^−1^.

### 2.7. Lipid Peroxidation

Lipid peroxidation was done by the method of Utley et al. [23]. Briefly, 1 mL of homogenized brain tissue was incubated in a metabolic shaker at 37 °C for one hour, 1.5 mL of 20% TCA was added, which was then centrifuged at 600 g for 10 min. Next, 1 mL of supernatant was added to 1 mL of freshly prepared Thiobarbituric acid (0.67%). The reaction was kept in the water bath for 10 min. Upon cooling, absorbance was read at 535 using a reagent blank. Values were expressed as nanomoles of malondialdehyde formed hour^−1^ mg protein^−1^.

### 2.8. Ascorbic Acid (AsA)

Ascorbic acid was determined by the method of Jagota and Dani [24]. First, 0.2 mL of homogenized brain tissue was treated with 0.8 mL of 10% TCA. After vigorous shaking, tubes were kept in an ice-cold bath for 5 min and centrifuged at 1200 g for 5 min. Next, 0.2 to 0.5 mL of the supernatant were diluted to 2 mL with distilled water, and 0.2 mL of Folin reagent (0.2 M) was added. After 10 min, the absorbance was read at 760 nm against a reagent blank. The amount of ascorbic acid was calculated from the standard graph. Values were expressed as μg of ascorbic acid μg protein^−1^.

### 2.9. Reduced Glutathione (GSH)

The estimation was carried out by the method of Beutler et al. [25]. Briefly, 0.4 mL of homogenized brain tissue was mixed with 3.6 mL of double-distilled water and followed by treatment with 0.6 mL of precipitating reagent (containing 1.67 g of glacial metaphosphoric acid, 0.2 g of EDTA, and 30.0 g of NaCl and made up to 100 mL with double distilled water).

The above reaction mixture was centrifuged at 600 g for 10 min. To 0.3 mL of supernatant, 2 mL of Na_2_HPO_4_ (0.3 M) and 0.25 mL of 5,5′ dithio-bis-2-nitrobenzoic acid (0.4% in 1% sodium citrate) were added, and the volume was made up to 3 mL with DDW. OD was read at 412 nm against blank. Values were expressed as μg of reduced glutathione μg protein^−1^.

### 2.10. In Silico Testing of Quercetin Toxicity and Molecular Docking

The VirtualToxLab™ is an online platform to estimate the toxicity of drugs, which requires the test compound to be submitted in pdb format. Therefore, the quercetin structure was downloaded from PubChem (PubChem CID: 5280343) in sdf format and was converted to the pdb format using the discovery studio software. The interaction of quercetin with the glucocorticoid, estrogen α, estrogen β, androgen, aryl hydrocarbon, thyroid α, thyroid β, mineralocorticoid, progesterone, hERG, liver X, and PPARγ was evaluated. In addition to these proteins, the enzymes cytochrome P450 1A2, 2C9, 2D6, and 3A4 were assessed for their interactions with quercetin. Flexible molecular docking was conducted for the quercetin and the moderately bound proteins in the VirtualToxLab running on an automated protocol. The low-energy poses are sampled into a dataset. The binding affinities between the quercetin and the target proteins were quantified using the dataset as input for Boltzmann scoring (Software BzScore4D) [16]. The binding mechanisms with these biomolecules were used to assess the protective mechanisms of quercetin to CCL4 toxicity in the brain.

## 3. Results

**Figure 1:**
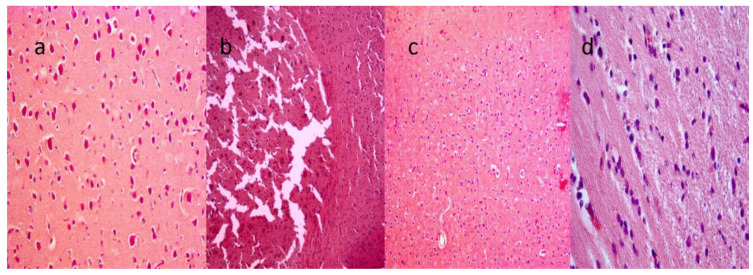
Cortex sections of normal and treated groups at a scale bar of 100 µm. (**a**) Normal histological appearance of brain tissues with neurocytes having well-defined nuclei. (**b**) CCL4-treated brain cortex section with widespread intracellular vacuolization and infiltration of inflammatory cells (aster). Neurocytes have dark eosinophilic cytoplasm, with cells having heterochromatic nuclei. (**c**) Quercetin-treated brain tissue has fewer vacuoles and inflammation. (**d**) Q + CCL4-treated brain section with mild vacuolization and mild infiltration of inflammatory cells (*n* = 3).

CCL4 is lipophilic and passes freely through all biological membranes, including the brain′s myelin sheath. A marked effect on brain tissue histology was observed with the cortex showing swelling, vacuolar degeneration, and karyopyknosis. All these changes are morphological characteristics of apoptosis. Figure 1a is the normal histology of the cerebral cortex of the brain. Extensive vacuolization was seen in myelin within the white matter, and a few vacuoles were also seen in the gray matter of rats treated with CCL4. CCL4-treated neurons had large nuclei turning from basophilic to pyknotic revealing apoptosis (Figure 1b). However, the normal histology was observed in quercetin-treated rats (Figure 1c). Figure 1d shows a complete reversal of altered histology in rats treated with quercetin two hours before CCL4.

**Figure 2:**
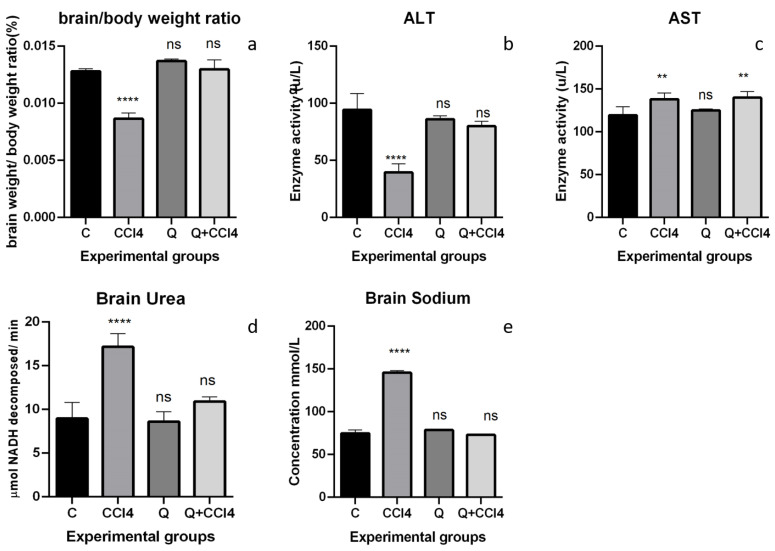
(**a**–**e**). Effect of CCL4 (1 mg/kg) and Q (100 mg/kg b.w) on the brain-to-body weight ratio, ALT, AST, brain urea, and brain sodium (*n* = 6). Data were analyzed by (one-way ANOVA), and Tukey’s test was used for multiple comparisons. Treated groups are compared to the control group. **** *p* < 0.0001, ** *p* < 0.01, ns is non-significant.

The effect of CCL4 on the brain-to-body weight ratio is presented in Figure 2a. The brain-to-body weight ratio decreased significantly (*p* < 0.0001) in the CCL4 treatment group, showing this group′s metabolic or growth disorders. Treatments with quercetin and combination groups completely reversed these groups′ metabolic or growth disorders and showed non-significant differences with control group growth patterns.

Figure 2b shows significantly decreased levels of ALT in group CCL4-treated rats (*p* < 0.0001) when compared to Group C (control). Quercetin treatment alone (Group Q) caused a non-significant decrease in the ALT levels (*p* > 0.05) compared to controls. Pretreatment of quercetin in Group IV reversed the decreased ALT levels and showed significant protection when compared with Group CCL4. The decreased ALT levels in the group Q + CCL4 were non-significant (*p* > 0.05) compared to the control group.

Figure 2c shows significantly increased levels of AST in Group CCL4 rats (*p* < 0.0001) when compared to control, indicating the induction of brain damage as AST catalytic activities in the CSF are linked to high risk. Quercetin treatment alone was relatively similar to control in the AST levels (*p* > 0.05) compared to control. However, pretreatment of quercetin in the Q + CCL4 group could not reverse the increased levels of AST.

Figure 2d shows significantly increased levels of urea in Group CCL4 rats (*p* < 0.0001) when compared to control, indicating brain damage and increased metabolic activity. Quercetin treatment alone caused a non-significant increase in urea levels compared to controls. However, pretreatment of Quercetin in the Q + CCL4 group decreased the increased urea levels significantly.

Figure 2e shows significantly increased sodium levels in the group Q rats (*p* < 0.0001) compared to controls, which is another indication of brain damage due to the voltage potential. Quercetin treatment alone was relatively similar to control; non-significant change in the sodium levels (*p* > 0.05) compared to controls. Pretreatment of quercetin in the Group Q + CCL4 reversed the increased sodium levels and showed significant protection compared with the group CCL4. The increase of sodium levels in group Q + CCL4 was significantly decreased (*p* < 0.001) compared to the control group, relatively similar to controls.

Table 1 shows the levels of biochemical oxidative stress biomarkers lipid hydroperoxides, ascorbic acid, and reduced glutathione catalase and superoxide dismutase in control and experimental rats (Table 1). The levels of lipid hydroperoxides and AsA were significantly (*p* < 0.05) increased in CCL4- treated rats compared to control and quercetin groups. The treatment of rats with quercetin resulted in significant recovery of these free radicals generated in brain tissues of group Q + CCL4. Exposure with CCL4 significantly decreased the reduced glutathione (*p* < 0.05) that was reversed considerably in group Q + CCL4 (*p* < 0.05). Catalase activity was reduced substantially with CCL4 treatment that was significantly recovered by treatment with quercetin in group Q + CCL4 (Table 1).

**Figure 3:**
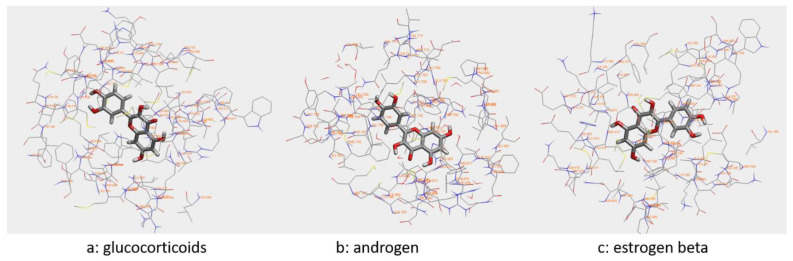
Docking conformation of quercetin with different targets. (**a**) Predicted bonded interactions (blue dashed lines) between quercetin and glucocorticoid; (**b**) binding interaction between quercetin and androgen; (**c**) binding interaction of quercetin and estrogen alpha. The ligand is based on atom type and the protein-based on amino acid residue type coloring.

The results from the VirtualToxLab™ are presented in Table 2. Quercetin was found to have a strong binding affinity to the androgens and had moderate binding to glucocorticoid and estrogen beta receptors. In addition, the quercetin was found to have weak binding to Estrogen receptor α (ERα), hERG, Peroxisome Proliferator-Activated receptor γ (PPARγ) and Thyroid receptor β (TRβ). Furthermore, quercetin did not bind to Aryl hydrocarbon receptor (AhR), Thyroid receptor α, Mineralocorticoid receptor (MR), progesterone receptor (PR), hERG, and Liver X receptor (LXR), nor to any of the cytochrome P450 enzymes. Figure 3 shows real-time 3D/4D visualization of binding modes of quercetin with glucocorticoids, estrogen beta, and androgen receptors in concomitance with binding results from BzScore4D. Based on the results, a possible mechanism of quercetin protection against CCL4 toxicity in the rat brain was hypothesized.

## 4. Discussion

CCL4 is a lipophilic colorless liquid and readily crosses cell membranes, including the blood–brain barrier. All body tissues rapidly take up CCL4, but the toxicity on the brain remains poorly understood. Some studies reported that adverse events of CCL4 in rodents are mainly based on systemic effects in the liver (centrilobular necrosis) and some impact on the kidney in inducing free radical toxicity and some tissues injuries [26]. However, in this study, we evaluated all the parameters in the brain tissue itself. In this study, the elevation of ROS was reported in brain cells on exposures to a single dose of CCL4 that is scavenged by antioxidant quercetin during normal cell metabolism. Histopathological analysis on brain tissues indicated a CCL4 induced karyopyknosis, apoptosis, and swelling. The toxic effect of CCL4 is believed to be due to trichloromethyl radicals produced during oxidative stress, as per previous literature [27]. The results of our study are in corroboration with Ritesh et al., 2015, who reported that a single hepatotoxic dose of CCL4 is equally neurotoxic to rats as many consecutive doses [5]. Organ and body weights showed significantly decreased growth changes in the brain-to-body weight ratios. The brain/bw ratio is one of the most sensitive parameters measured for detecting the effect of exposure to toxins on growth and development [28]. CCL4 significantly altered AST and ALT activity in brain tissue, and no modified ALT levels were observed in the prophylactically quercetin-treated group. CCL4 treatment causes brain tissue damage followed by the release of AST molecules into the extracellular spaces of brain tissue. The function of this enzyme is the reversible transport of amines from aspartate to α-ketoglutarate [29]. In concomitance to this study, Kelbich et al. reported an increase in AST catalytic activities in the cerebrospinal fluids of cerebral ischemia patients [30].

Furthermore, the increased AST activity could be connected with increased transport of NADH from the cytosol to mitochondria. In contrast, the increased ALT activity would represent more transformation of pyruvate to alanine due to increased glycolysis and hence increased pyruvate [31]. Increased brain urea by CCL4 was reversed by quercetin. The ‘reverse urea effect causes brain edema’, i.e., the significant urea gradient between blood and brain causes an inflow of water into the brains of induced animals [32]. For the first time, this study has evaluated brain sodium levels with CCL4 exposure. Brain sodium is increased with sympathoexcitatory and pressor responses [33], which further destroys brain potential and leads to brain inflammation. In addition, increased sodium levels hyperactivate Na/K channels that trigger excitotoxic neuron death [34]. In concomitance to sodium levels, the Herg k^+^ channel (Pottasium channel) was found to show weak binding with quercetin in silico analysis. Hence, it can be predicted that quercetin protected via the action of Na^+^/K^+^ pump. However, the action of protection by quercetin with respect to Na^+^/K^+^ pump must be verified in future studies. Glutathione eliminates reactive oxygen species (ROS) produced in oxidative stress [35]. GSH, a ubiquitous intracellular cytosolic tripeptide at millimolar concentrations, is the primary non-enzymatic biomarker of redox homeostasis. The reduced GSH in brains after the exposure to CCL4 could result from increased GSH-peroxidase activity in the exposed rat. The decreased GSH was further associated with ascorbic acid and lipid peroxidation [19,36,37]

Additionally, glutathione depletion induces glycogenolysis-dependent ascorbic acid synthesis in murine hepatocytes in vitro [38]. The significant decreased levels of antioxidant enzymes such as CAT and SOD were reversed to normal by quercetin supplementation (Table 1). Based on our results, we conclude that quercetin shows significant prophylactic effects against CCL4 with respect to the studied parameters. Thus, it was indicated that quercetin prevents alterations in oxidative stress parameters and neurotransmitters parameters [39]. Our results highlight the importance of understanding the potential prophylactic effects of quercetin against neurotoxicity.

Based on the binding affinities of quercetin to various proteins, the normalized binding affinity of quercetin to the studied proteins was found to be 0.418 (average). It should be noted that the toxicity values can be overestimated since binding to a particular protein may or may not lead to any adverse event. The software used for the flexible docking in VirtualToxLab is Alignator and Cheetah. All the binding modes between the quercetin and the target proteins were identified during the study. The interaction between quercetin and the target proteins, which showed moderate binding, is given in Figure 3. The binding affinity are defined between >100 μM (not binding) to <1.0 nM (strong binding). The overall binding potential ranged from 0.0 (benign) to 1.0 (extreme) [16].

Moderate binding between quercetin and glucocorticoids, estrogen beta, and androgens was evaluated. Previous studies suggested that oxidative stress increases glucocorticoid (GC) hormones; hence, the hippocampus, which has a high concentration of GC receptors, is especially vulnerable to elevated levels of GCs. The GCs have been suggested to endanger hippocampal neurons by exacerbating the excitotoxic glutamate-calcium-reactive oxygen species (ROS) cascade [40,41,42]. Our binding results suggest quercetin binding to glucocorticoids as a preventing mechanism for preventing ROS cascade generated by CCL4 neurotoxicity.

Previous studies demonstrated that estrogen receptor β (ERβ) signaling alleviates systemic inflammation in animal models, and suggested that ERβ-selective agonists may deactivate microglia and suppress T cell activity via the downregulation of nuclear factor ĸ-light-chain-enhancer of activated B cells (NF-ĸB) [43]. The estrogen receptor agonists play an essential role in protecting the central nervous system against neuroinflammation and neurodegeneration. Quercetin aglycone and its glucuronide possess estrogenic activity, and quercetin is also classified as a phytoestrogen [8,44]. Thus, the binding of quercetin to estrogen alpha and beta might have caused the protection against the stress induced by carbon tetrachloride [8]. Future studies are needed to verify this mechanism for protection. Another possible mechanism for the protection of quercetin can be the suppression of stress-induced plasma corticosterone and adenocorticosterone levels. The DNA binding activity of the glucocorticoid receptor is modulated on binding to quercetin and is one of the possible reasons for suppressing plasma corticosterone and adrenocorticotropic hormone levels [45,46]. The effect of androgens on the cerebral vasculature is a complex mechanism. They have both protective and detrimental effects, depending on several factors, such as age, drug dose, and state of disease. Chronically elevated androgens are pro-angiogenic, promote vasoconstriction, and influence blood–brain barrier permeability. In addition to these, androgens have been shown to affect the cerebral vasculature [44] directly. This study found moderate binding with androgens; hence, we propose that elevated androgen levels by CCL4, which could otherwise be harmful to the brain, were probably prevented by quercetin by binding with androgen receptors. Future studies are needed to verify our hypothesis.

## Figures and Tables

**Table 1:** Effect of quercetin on CCL4 induced oxidative stress in the brains of control and experimental rats.

Treatment Groups	Control	CCL4 (1 mg/kg)	Q (100 mg/kg)	Q + CCL4
**Lipid Peroxidation** (mmoles of malonaldehyde formed/hour/mg protein)	1.98 ± 0.44 ^b,d^	8.33 ±0.82 ^a,c,d^	0.94 ± 0.15 ^b,c^	0.83 ± 0.35 ^a,b,c^
**Ascorbic acid** (µg of ascorbic acid/µg protein)	0.73 ± 0.15 ^b,d^	3.23 ± 1.82 ^a,c,d^	0.45 ± 0.06 ^b,d^	1.70 ± 0.14 ^a,b,c^
**Reduced glutathione** (µg of GSH/mg protein)	5.30 ± 0.80 ^b^	2.53 ± 1.47 ^a,c,d^	5.55 ± 3.47 ^b^	6.90 ± 0.41 ^b^
**Catalase** mmol H_2_O_2_/min/mg protein	3.05 ± 0.55 ^b,c^	0.26 ± 0.30 ^a,c,d^	2.30 ± 0.45 ^a,b,d^	2.43 ± 0.85 ^b,c^
**Superoxide dismutase** Units/mg protein	135.29 ± 44.79 ^b^	63.11± 22.48 ^a,d^	131.26 ± 7.29 ^d^	126.10± 25.73 ^b,c^

Querectin (100 mg/kg b.w) was administered 2 h before CCL4 assault. Data are representative of mean ± SD of three independent experiments, each group containing six mice. ^a^ significant (*p* < 0.05) when compared to control; ^b^ significant (*p* < 0.05) when compared to CCL4 (1 mg/kg) group; ^c^ significant (*p* < 0.05) when compared to Q (100 mg/kg) group; ^d^ significant (*p* < 0.05) when compared to Q + CCL4 group.

**Table 2:** Binding of quercetin to various proteins.

Target	Binding Type	Binding Affinity (VirtualToxLab)
Androgens	moderate binding	948 nM
Aryl hydrocarbon	negligible	>100 μM
CYP1A2	negligible	>100 μM
CYP2C9	negligible	>100 μM
CYP2D6	negligible	>100 μM
CYP3A4	negligible	>100 μM
Estrogen α,	weak binding	3.54 μM
Estrogen β	moderate binding	448 nM
Glucocorticoid	moderate binding	574 nM
Herg k^+^ channel	weak binding	4.87 μM
Liver x	weak binding	72.7 μM
Mineralocorticoid	weak binding	14.7 μM
Pparγ	weak binding	4.53 μM
Progesterone	weak binding	37.2 μM
Thyroid α,	weak binding	15.3 μM
Thyroid β	weak binding	5.85 μM

Overall toxic potential was found to be 0.418 [16].

## Data Availability

Data will be available on request to corresponding author.
